# A Clonal NG2-Glia Cell Response in a Mouse Model of Multiple Sclerosis

**DOI:** 10.3390/cells9051279

**Published:** 2020-05-21

**Authors:** Sonsoles Barriola, Fernando Pérez-Cerdá, Carlos Matute, Ana Bribián, Laura López-Mascaraque

**Affiliations:** 1 Departamento de Neurobiología Molecular, Celular y del Desarrollo, Instituto Cajal-CSIC, 28002 Madrid, Spain; sonsolesbarriola@cajal.csic.es (S.B.); abribian@cajal.csic.es (A.B.); 2 Centro de Investigación Biomédica en Red de Enfermedades Neurodegenerativas (CIBERNED), Departamento de Neurociencias, Universidad del País Vasco, 48940 Leioa, Spain; fernando.perez@ehu.eus (F.P.-C.); carlos.matute@ehu.eus (C.M.); 3 Achucarro Basque Center for Neuroscience, 48940 Leioa, Spain

**Keywords:** NG2-glia, progenitors, multiple sclerosis, lineage, in utero electroporation, morphometric analyses, clonal analyses, lesioned brain

## Abstract

NG2-glia, also known as oligodendrocyte precursor cells (OPCs), have the potential to generate new mature oligodendrocytes and thus, to contribute to tissue repair in demyelinating diseases like multiple sclerosis (MS). Once activated in response to brain damage, NG2-glial cells proliferate, and they acquire a reactive phenotype and a heterogeneous appearance. Here, we set out to investigate the distribution and phenotypic diversity of NG2-glia relative to their ontogenic origin, and whether there is a clonal NG2-glial response to lesion in an experimental autoimmune encephalomyelitis (EAE) murine model of MS. As such, we performed in utero electroporation of the genomic lineage tracer, StarTrack, to follow the fate of NG2-glia derived from single progenitors and to evaluate their response to brain damage after EAE induction. We then analyzed the dispersion of the NG2-glia derived clonally from single pallial progenitors in the brain of EAE mice. In addition, we examined several morphological parameters to assess the degree of NG2-glia reactivity in clonally-related cells. Our results reveal the heterogeneity of these progenitors and their cell progeny in a scenario of autoimmune demyelination, revealing the ontogenic phenomena at play in these processes.

## 1. Introduction

Multiple sclerosis (MS) is a chronic, disabling autoimmune and neurodegenerative disorder targeting the white and gray matter of the central nervous system (CNS) [1,2,3]. The loss of myelin and oligodendrocytes, axonal loss, and glial scar formation in the brain are the hallmarks for MS. As such, much interest has been generated by NG2-glia (also referred to as oligodendrocyte progenitor cells (OPCs) due to their ability to form myelin and the fact that they undergo a reactive response to brain injury. These cells are the fourth-most distinct major glial cell population [4], and they constitute 8–9% of the total cells in the white matter and 2–3% of the total cells in the grey matter [5]. Moreover, they express the NG2 chondroitin sulfate proteoglycan (neural/glial antigen 2 -NG2), the alpha receptor for platelet-derived growth factor (PDGFRα), as well as other oligodendrocyte markers. NG2-glia also have several distinguishing physiological properties, expressing voltage-gated ion channels and participating in neural–glial interactions [6,7,8]. They are thought to fulfil diverse functions in the brain, and they are implicated in the development of the nervous system, in maintaining its homeostasis, in neuromodulation, and in sustaining (re-)myelination [9,10,11,12]. The importance of these cells for the proper functioning of the brain is also reflected in the fact that they are distributed throughout the entire brain and that a constant pool of NG2-glia is maintained in the brain [13,14].

In animal models used to probe CNS lesions, NG2 cells proliferate and migrate to the site of injury, where they differentiate into oligodendrocytes [15,16,17]. In response to brain injury, some NG2-glia change their morphology and adopt a reactive phenotype, like astrocytes [18,19,20]. This morphology is characterized by thickened and highly ramified processes, as opposed to the relatively thin and unbranched prolongations of non-reactive NG2-glia [17]. Furthermore, NG2-glia display both molecular and behavioral heterogeneity following brain injury [17,21,22], producing both beneficial and deleterious effects [8,21,23,24,25,26,27]. However, little is known about the existence of certain subsets of NG2-glia that produce a specific response to brain damage and whether their fate may already be determined early in embryonic development.

A population of NG2-glia are known to arise from embryonic neural progenitor cells (NPCs) located in the subpallium [28]. Moreover, NG2-glia that come from dorsal NPCs (pallium) migrate following radial glia processes and group in clones throughout the cortex [13,29,30]. Thus, it is essential to track individual NPCs, and to achieve heritable and stable labelling of their progeny. Here we used the StarTrack approach, a reliable and proven method for clonal analysis based on the random genomic integration and expression of twelve fluorescent proteins [18]. In this study, we focused on the progeny of NG2-glia derived from individual embryonic glial fibrillary acidic protein-expressing ￼￼ progenitors (GFAP^+^) in the brain of mice in which experimental autoimmune encephalomyelitis (EAE) was induced. Employing StarTrack in in these EAE-lesioned mice allowed us to analyze the clonal distribution and morphological cell response of NG2-glia in EAE lesions. Accordingly, we performed a morphometric analysis on the progeny of these cells, evaluating different parameters to assess the morphological changes to NG2-glia in the EAE mouse model. This analysis enabled us to assess the intensity of NG2-glia activation and to identify the heterogeneity in the phenotypes, clonal size, and cell fate of the individual progenitors singled out by the StarTrack approach.

## 2. Materials and Methods

### 2.1. Animals

C57BL/6 mice employed in this study were the same as those previously used to analyze the clonal response of astrocytes in a murine model of MS [19]. In brief, mice from Janvier Labs were housed in standard cages at the Universidad del País Vasco (UPV)-EHU animal facility, maintained on a 12 h controlled light–dark cycle with food and water available ad libitum. The study was carried out in accordance with the European Union recommendations on the use and welfare of experimental animals (2010/63/EU), and those of the Spanish Ministry of Agriculture (RD 1201/2005 and L 32/2007). The Bioethical Committee at the UPV-EHU approved the protocol. Nine adult StarTrack-electroporated mice (three sham and six EAE mice) were used. Clones were analyzed in all EAE mice. Animals that displayed NG2-glial clones—three of the EAE-lesioned mice (two males and one female)—were selected for the clonal analysis of NG2-glia. For the morphometric analysis, a selection of ten cells from EAE-lesioned mice and six cells from sham was made.

### 2.2. StarTrack DNA Vectors

Clonal analysis was accomplished using the StarTrack approach. StarTrack DNA vectors were produced as described previously [13,19,31] and the StarTrack mixture consisted of twelve PiggyBac constructs containing the six fluorescent nuclear and cytoplasmic proteins driven by the GFAP promoter (XFP), along with the hyperactive PiggyBac transposase (CMW-hyPBase) construct. The fluorescent proteins produced by the DNA vectors were the yellow fluorescent protein (YFP), monomeric Kusabira Orange (mKO), mCerulean, mCherry, mT-Sapphire and enhanced green fluorescent protein (EGFP).

### 2.3. In Utero Electroporation (IUE)

IUE was performed as previously described [19]. Embryonic day (E)14 pregnant mice were anesthetized with 4% isoflurane (2 mL/L: Isova vet, Centauro), their uterine horns were exposed by midline laparotomy and the embryos they contained were visualized by trans-illumination with cold light. The plasmid mixture was injected into the lateral ventricle (LV) of each embryo, which then received one or two trains of five square pulses (35 V, 50 ms duration, 950 ms intervals). Finally, the uterine horns were placed back into the abdominal cavity and the electroporated embryos were allowed to continue their normal development until birth.

### 2.4. EAE Induction

Chronic, relapsing EAE was induced in C57BL/6-electroporated mice during the eighth post-natal week [19,32,33]. Each animal was first immunized with a subcutaneous injection (300 µL) of a 1:1 ratio of myelin oligodendrocyte glycoprotein antigen solution (MOG35-55 peptide, 200 ng/mouse: Sigma, Barcelona, Spain) and complete Freund’s adjuvant (CFA), followed by an intraperitoneal injection of pertussis toxin (500 ng: Sigma) in 100 µL of phosphate-buffered saline (PBS) on the day of immunization and two days later. CFA is a solution that contains *Mycobacterium tuberculosis* H37RA (8 mg/mL) in incomplete Freund’s adjuvant. EAE was scored double-blind each day: 0, no noticeable signs of EAE; 1, flaccid tail; 2, paralyzed tail; 3, impairment or loss of muscle tone in hindlimbs; 4, unilateral partial hindlimb paralysis; 5, total bilateral hindlimb paralysis; 6, complete hindlimb paralysis and loss of muscle tone in the forelimbs; 7, complete paralysis of the forelimbs and hindlimbs; and 8, moribund. In our experiments, the motor symptoms in mice with EAE initiated around 10 days’ post-immunization and progressively aggravated until reaching a peak typically at day 21, and declined slightly thereafter during the chronic phase [32]. EAE was successfully induced in all mice used in this study, and the scores representing the symptoms of the three EAE mice were 1.75 (nearly paralyzed tail), 3, and 4.5 (see Figure 2C from Bribián et al., 2018 [19]). Since tissue damage and demyelination parallels the symptoms, we assumed that the NG2-glial clonal response was maximal at that peak of the symptoms and accordingly, analyzed brain tissues at that stage. Results between animals were homogeneous.

### 2.5. Immunohistochemistry

Mice were perfused 21 days’ post-induction (dpi) with 4% paraformaldehyde (PF) in a phosphate buffer (PB). They were then post-fixed for over 2 h in the same solution and stored at 4 °C in PBS. Coronal vibratome sections (50 µm) were washed and permeabilized three times with 0.5% Triton X-100 (PBS-T), washed three times in 0.1% PBS-T, and blocked for 30 min at room temperature (RT) with 5% normal goat serum (NGS, S26-100ML: Merck-Millipore). Brain sections were incubated overnight at 4 °C with the following antibodies in 5% NGS and 0.1% PBS-T: rabbit anti-PDGFRα (1:300, 3174S: Cell Signaling) and biotinylated tomato lectin (TL, 1:50, L0651: Sigma-Aldrich). After washing the brain slices three times with 0.1% PBS-T, they were incubated for 2 h at RT with a secondary antibody coupled to Alexa 633 (1:1000, Invitrogen) or a Streptavidin–Alexa Fluor 633 conjugate (1:1000, S21375: Invitrogen Life Technologies (Carlsbad,. CA, USA ). Prior to visualization, they were washed 6 times in 0.1% PBS-T and then 1× PBS.

### 2.6. Imaging Acquisition and Data Analysis

The expression of the different fluorescent proteins was first checked under an epifluorescence microscope (Nikon, Eclipse F1) equipped with filters (Semrock) optimized for the following fluorophores: YFP (FF01-520/15), mKO (FF01-540/15), Cerulean (FF01-405/10), mCherry (FF01-590/20), Cy5 (FF02-628/40-25), GFP (FF01-473/10), and UV-2A (FF01-334/40-25). Consequently, images were acquired on a confocal microscope (Leica, TCS-SP5) and the emission for each fluorescent protein was obtained in separated channels using different excitation (Ex) and emission (Em) wavelengths (in nanometers, nm): mT-Sapphire (Ex: 405; Em: 520–535), mCerulean (Ex: 458; Em: 468–480), EGFP (Ex: 488; Em: 498–510), YFP (Ex: 514; Em: 525–535), mKO (Ex: 514; Em: 560–580), mCherry (Ex: 561; Em: 601–620), and Alexa 633 (Ex: 633; Em: 650–760). Laser lines were situated between 25% and 40%, and maximum projections were obtained using the confocal (LASAF Leica) and NIH-ImageJ software. Affected or lesioned areas were localized by TL staining and the perimeters of the lesion site were defined using the “enlarge” tool of NIH-ImageJ software, with a distance of 50 µm between the concentric perimeters. The Simple Neurite Tracer (SNT) plugin (NIH-ImageJ) [34] and a Scholl analysis [35,36] were used for the morphological analysis. The statistical analysis of the data and the graphical representations were performed using the R statistical software package (version 3.5: R Core Team, 2018), and the Prism 5 (GraphPad) software. Statistical significance was evaluated using either a two-tailed unpaired Student’s t test for 2-group comparisons or a one-way ANOVA followed by Dunnett’s post hoc test for multiple group comparisons. Values with a confidence interval of 95% (*p* < 0.05) were considered statistically-significant and significant differences between the groups are indicated in the graphs with asterisks: * *p* < 0.05, ** *p* < 0.01, *** *p* < 0.001.

## 3. Results

### 3.1. Spatial Distribution of the Cortical Progeny of NG2-Glia Derived from Single Embryonic Progenitors in EAE-Lesioned Mouse Brain

**Figure 1:**
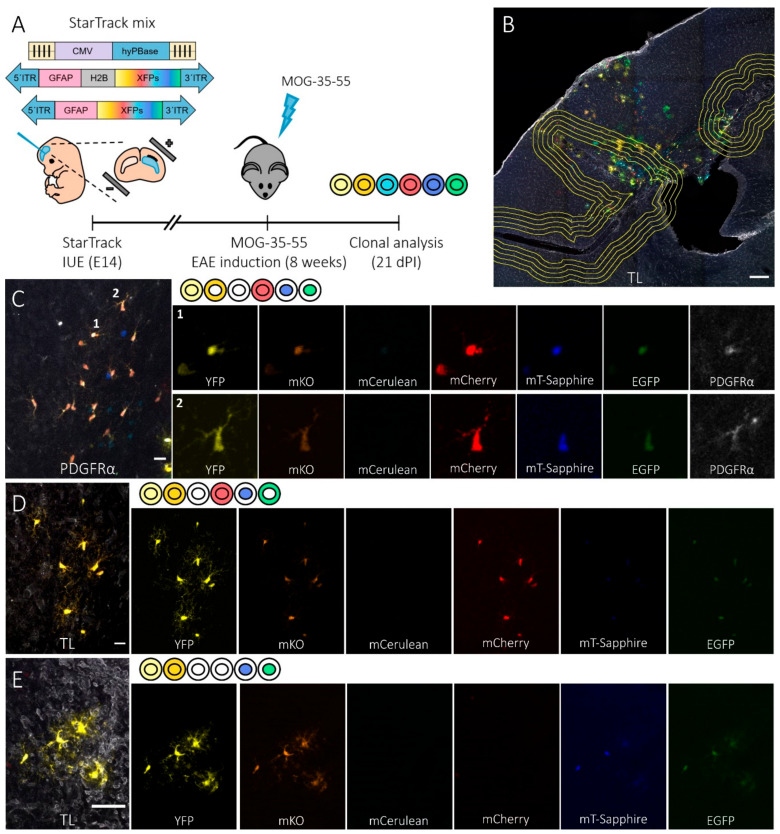
StarTrack-labelled NG2-glia progeny of individual dorsal subventricular zone (SVZ) progenitors after the induction of EAE. (**A**) Time-line showing the experimental approach: in utero electroporation (IUE) of the StarTrack mix into E14 dorsal neural progenitor cells (NPCs) in the lateral ventricle (LV), induction of experimental autoimmune encephalomyelitis (EAE) in mice eight weeks after electroporation, and clonal analysis 21 days’ post-induction (dpi). The StarTrack mixture contained a hyperactive PiggyBac transposase (hyPBase) and 12 plasmids that encoded six different fluorescent proteins (XFPs) expressed in either the nucleus or cytoplasm. A glial fibrillary acidic protein (GFAP) promoter drove XFP expression. (**B**) Coronal view of the brain showing the location of EAE lesions in the cerebral cortex, corpus callosum, and striatum, as identified by tomato lectin (TL) labelling. A line surrounding the lesion core and four concentric compartments separated by 50 µm was used to measure the distance of the NG2-glia sibling cells from the lesion. StarTrack-labelled both astrocytes and NG2-glia around the different lesion sites. (**C**–**E**) Detail of the coronal views of sibling NG2-glia dispersed throughout the corpus callosum (**C**) and cerebral cortex (**D**,**E**). The color-codes and the corresponding XFPs expressed are detailed at the side of the merged images. NG2-glia were identified by platelet-derived growth factor (PDGFRα^+^) staining (**C**: 1 and 2), TL was used to identify the EAE affected and lesion areas (**E**), and the non-affected zones (**D**). Scale bars, 200 µm (**B**) and 20 µm (**C**–**E**).

To evaluate how the NG2-glia progenitors responded to EAE lesions in the phase of symptom improvement, we combined the genomic StarTrack tool with the induction of EAE in mice. We first targeted individual E14 dorsal (pallial) progenitors in the LV (Figure 1A) [13]) through IUE of the StarTrack mixture (driven by the GFAP promoter) with the hyperactive PiggyBac transposase (CMW-hyPBase: Figure 1A). Since up to 4096 different color-code combinations can be achieved with this method, it facilitates a precise clonal analysis of the progeny of single labelled cells with an active GFAP promoter [37]. Eight weeks after birth, the MOG peptide was administered to analyze the clonal NG2-glia response to EAE lesions 21 dpi (Figure 1A).

**Figure 2:**
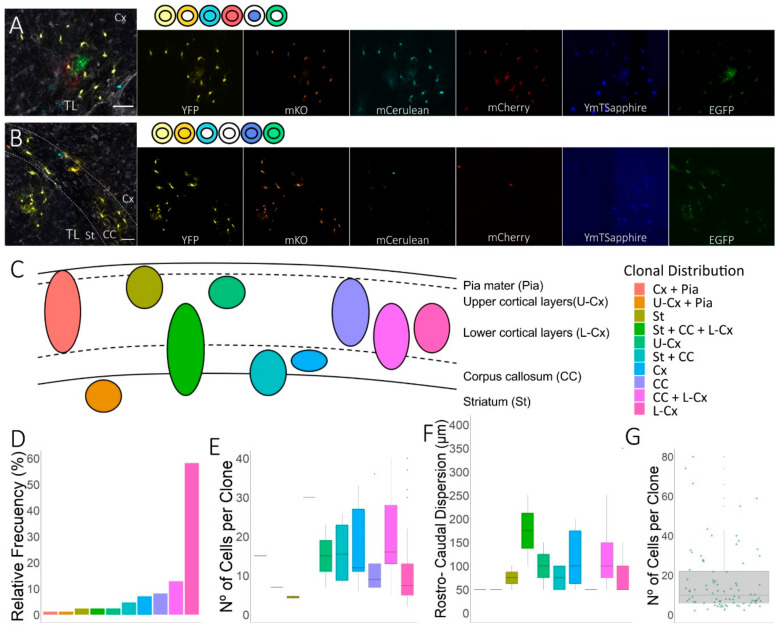
Clonal distribution of NG2-glia in coronal sections. (**A**,**B**) Detail of sibling NG2-glia close to the lesion in the cerebral cortex (**A**) and striatum and corpus callosum (**B**). Affected areas were identified by tomato lectin (TL) staining and clonally-related cells showed equivalent color combinations, detailed at the side of the merged images. (**C**) Scheme showing the diverse patterns of clonal NG2-glia dispersion. Dorsal E14 progenitors from the lateral ventricle produced different patterns of clonal NG2-glia progeny in the EAE-lesioned brain, with a heterogeneous clonal dispersion within the striatum (St), corpus callosum (CC), the upper- (U-Cx] and lower-cortical layers (L-Cx]), and the pia mater (Pia). (**D**) The relative frequency of the different clonal distributions estimates the frequency of each clone pattern in the EAE mice, indicating that NG2-glia clones derived from pallial progenitors expanded preferentially within the lower cortical layers. (**E**) The number of cells per clone varied depending on the cell fates of the clone. The largest clones were those distributed within the CC. (**F**) The largest clones exhibited a more extended rostro-caudal axis dispersion with sibling cells occupying a maximum average dispersion of 330 µm. (**G**) The clones varied in size from 3 to 80 cells, with an average of 17 cells per clone. Scale bars, 50 µm (**A**,**B**).

To perform the clonal analysis of NG2-glia in EAE mice, cortical regions containing StarTrack-labelled cells close to affected areas were selected (Figure 1B). These areas were characterized by the presence of perivascular inflammatory infiltrates and enlarged perivascular spaces revealed by TL staining (Figure 1B). This TL staining allowed a perimeter to be drawn around the core of the lesions in both the cortex and striatum. To analyze labelled cells in the affected area, the perimeter surrounding the lesion core was amplified four times using the “enlarge” tool (NIH-ImageJ), obtaining borders at 50 µm, 100 µm, 150 µm, and 200 µm (Figure 1B). Clonal dispersion of the sibling astrocytes and sibling NG2-glia close to the lesion sites (Figure 1B,E) was identified in the cortical layers (Figure 1B,D,E, and Figure 2A), the corpus callosum (Figure 1C and Figure 2B), and in part of the striatum (Figure 2B). The identity of the NG2-glia was confirmed through the expression of PDGFRα (Figure 1C) and sibling NG2-glia were identified by their uniform fluorescent color-code (i.e., the expression and localization of the XFPs). While some NG2-glia clones were distributed within or in close proximity to the lesion site (Figure 1D), others were located far from the affected areas (Figure 1E). Thus, combining EAE induction and StarTrack we were able to evaluate the clonal response of NG2-glia in an inflammatory and lesioned mouse brain.

### 3.2. Clonal Distribution of the Cell Progeny from Individual Embryonic Pallial NG2-Glia Progenitors in EAE-Lesioned Brains

We evaluated the clonal fate of NG2-glia generated from dorsal embryonic NPCs in the LV, and of clonally-related NG2-glia distributed within the cerebral cortex, corpus callosum, and striatum (Figure 1B and Figure 2). Sibling NG2-glia form clusters of up to 80 cells in lesioned brains, with clones having an average size of 17 cells (*n* = 84 analyzed clones, 1393 NG2-glial cells: Figure 2G). NG2-glia clones were distributed sparsely in specific regions within these three areas (Figure 2C), with different patterns of dispersion relative to the astrocyte clones [31]. Thus, we identified different patterns of NG2-glia clonal cell dispersion (Figure 2C), with clonally-related cells limited to the striatum, corpus callosum, or the upper or lower layers of the cortical cortex (Figure 2A). To obtain the relative frequency of each clone pattern, we calculated the number of times that each clone pattern appeared, divided by the total number of NG2-glia clones in the three EAE mice. Other clones expanded and colonized multiple regions of the brain (Figure 2B) but interestingly, none of the NG2-glia clones exclusively populated the pia matter (Figure 2D). As mentioned above, some NG2-glia clones were located in the striatum, with clonally-related cells situated in both the corpus callosum and lower cortical layers (Figure 2B,C). In addition, while 8% of the total clones were confined to the corpus callosum, 13% of NG2-glia clones populated both the corpus callosum and the lower cortical layers (Figure 2B,C,D). Nevertheless, most of the clones generated from NPCs in this EAE-lesion scenario (58% of the NG2-glia clones) were distributed in clusters found in the lower cortical layers (Figure 2A,D).

Interestingly, the average of NG2-glia clone size was larger when the clones colonized more than one region (Figure 2E). The distribution of clones with more cells corresponded to those clones in which some of the sibling cells were located in the corpus callosum. These larger clones populated both the striatum, the corpus callosum, and the lower cortical layers (Figure 2E). In addition, clones with cells in the corpus callosum showed the highest average spread in the rostro-caudal axis. NG2-glia clones were distributed in the striatum, the cortex, and pia, or just in the cortex where clones displayed the lower rostro-caudal dispersion (Figure 2F). This indicates that there was a correlation between rostro-caudal axis extension and the number of siblings NG2-glia cells per clone (Figure 2E,F).

Thus, NG2-glia clones derived from dorsal progenitors of the embryonic SVZ were distributed in ten different patterns of clonal dispersion, showing heterogeneity in relation to both their size and rostro-caudal distribution.

### 3.3. Relationship Between Sibling NG2-Glia and EAE Brain Lesions

**Figure 3:**
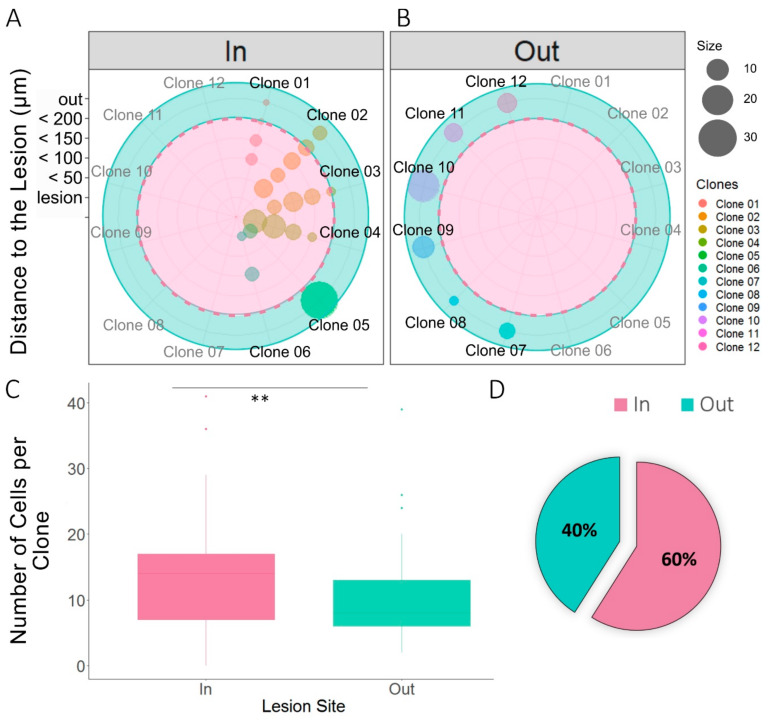
Dispersion of NG2-glia clones relative to the lesion. (**A**,**B**) Radial plots of sibling NG2-glia dispersion according to their distance from the EAE lesion and the number of clonally-related cells. The radii represent the lesion core area and the different consecutive perimeters (up to 200 µm) surrounding that area at an interval of 50 µm. All cells further than 200 µm away from the lesion core were considered to be outside of the lesion area (green area), and cells within 200 µm were considered to be inside the lesion area (pink area). Quadrants separate sibling cells of inner clones (01–06) (**A**) and from the outer clones (07–12) (**B**). Inner clones were described as clones with one or more clonally-related cells within at least 200 µm from the core. Sibling cells of six outer clones and six inner clones were represented, the sibling cells sharing the same color-code. (**C**) There were more clonally-related cells that formed clones within the lesion site (inner clones) than those that formed outer clones. Two-tailed unpaired Student’s t test was used to evaluate the statistically-significant differences between the two groups: ** *p* < 0.001. (**D**) From the NG2-glia clones analyzed (*n* = 48), 60% had at least one sibling cell within the 200 µm perimeter around the lesion. More inner clones than outer clones were found.

The clonal distribution of NG2-glia was evaluated by performing StarTrack clonal analysis in mice in which EAE lesions were induced. The NG2-glia clonal analysis revealed the distribution of different NG2-glia clones relative to the lesion. We quantified the number of sibling cells within the lesion core or in the four concentric perimeters (Figure 1B and Figure 3A), as well as the clonally-related cells outside the defining borders of the lesion (Figure 3B), analyzing 642 sibling cells from 48 different clones (Figure 3). Clones with cells inside the 200 µm perimeter around the lesion (Figure 3A) were considered as inner clones, whereas the rest were considered as outer clones (Figure 3B). We measured the distance from the lesion core of all sibling NG2-glia cells, showing a very heterogeneous dispersion (Figure 3A,B). Inner clones (Figure 3A) displayed multiple distribution patterns, the most characteristic was that in which there was an even dispersion of clonally-related cells around the lesion core (Figure 3A: clones 01–04, 06). Nevertheless, some inner clones were more widely dispersed, with sibling cells in and far from the lesion core (Figure 3A: clone 05). The average number of cells per inner clone was significantly higher than that of the outer clones (*p* < 0.001: Figure 3C). In addition, 60% of the analyzed clones analyzed were in close proximity to the lesion (Figure 3D).

### 3.4. Morphometric Analyses of NG2-Glia in EAE Lesions

**Figure 4:**
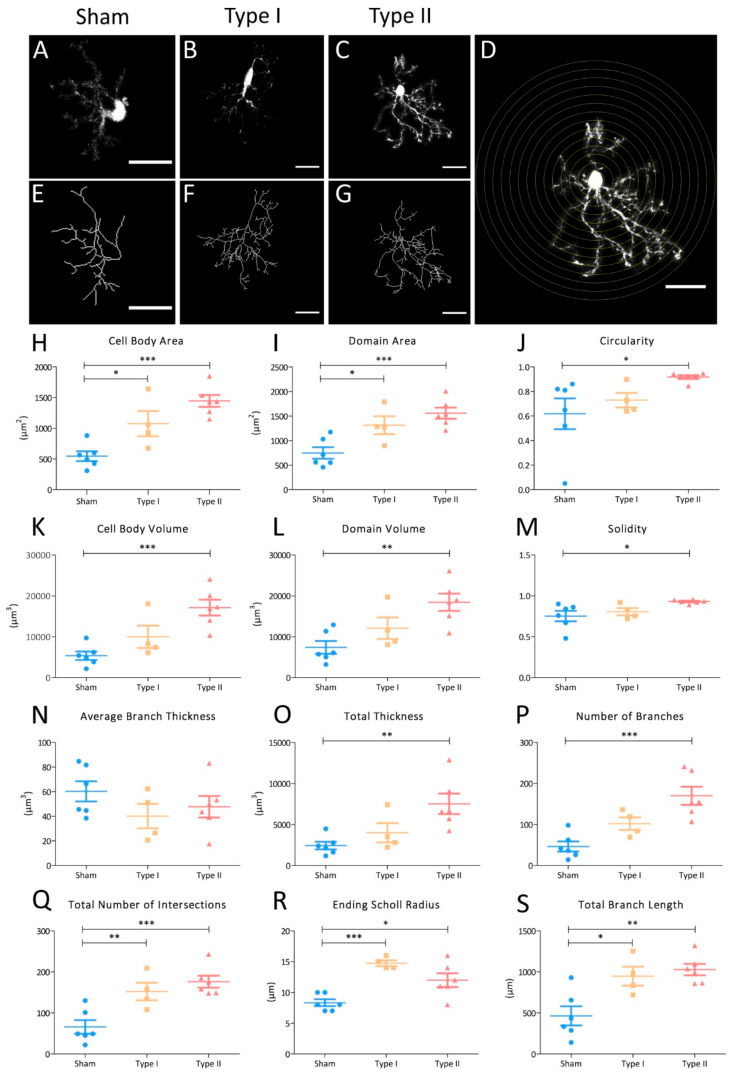
Morphometric parameters showing the shift of NG2-glia towards hypertrophy in the EAE brain. (**A**–**G**) Representative z-stack projection of StarTrack-labelled NG2-glia. Morphological analysis comparing sham NG2-glia (**A**,**E**) with both cell types identified in EAE mice: type I (**B**,**F**) and type II (**C**,**G**). Fill out images obtained once all paths were drawn using the Simple Neurite Tracer (SNT) plugin (NIH-ImageJ (**A**–**C**). Measurements of cell body area and volume, domain area and volume, circularity, solidity and thickness were acquired using this image. The respective rendered paths of the NG2-glia in filled out images (**E**–**G**). The number of branches were automatically measured from these rendered images. Representation of the Scholl analysis on a type II NG2-glia cell (**D**). The interval between each consecutive Scholl circle radius was 4 µm. The total number of intersections, total branch length and ending Scholl radius were obtained in this analysis. (**H**–**S**) Graphs of the parameters that describe the change in NG2-glia towards a more severe morphology. The morphology of type I and II NG2-glia was compared with that of sham NG2-glia. Statistically-significant differences across the groups were evaluated using a one-way ANOVA followed by Dunnett’s post hoc test for multiple group comparisons: * *p* < 0.05, ** *p* < 0.01, *** *p* < 0.001. Scale bars = 20 µm.

To analyze the cell heterogeneity in the morphological response of NG2-glia to EAE, we examined the StarTrack-labelled NG2-glia close to the lesion. Since NG2-glia react in response to brain damage, by thickening and branching their processes, we applied morphometric parameters to identify and quantify these changes. First, StarTrack-labelled NG2-glia within or far from the lesion core were divided into the two main subtypes, type I and type II cells, according to the primary branch thickness. The morphology of type I (*n* = 6 cells) and type II (*n* = 4 cells) was compared to that of sham NG2-glia (*n* = 6 cells) through both conventional morphological analysis and Scholl analysis with the SNT plugin (NIH-ImageJ: Figure 4D). Different cell parameters were analyzed to evaluate the progression of NG2-glia arborization in response to lesion (adapted from Yamada et al., 2013 [36]: Figure 4A–C,E–G): cell body area and volume, domain area and volume, circularity, solidity, average branch thickness, total thickness, number of branches, total number of intersections, ending Scholl radius, and total branch length. The cell body area and the domain area are 2D parameters (µm^2^) to measure the surface of the cell and the convex hull area from the projection image, respectively. The region enclosed by a polygon that connects the end-points of NG2-glia processes is considered as the convex hull area. The domain and cell body volume are 3D parameters (µm^3^) and consider the width of the cell, measuring the convex hull area and the cell body area rendered from z-series data sets, respectively. Both, domain area and volume, are parameters that indicate the tridimensional non-overlapping domain that NG2-glia occupy [5,14,38]. The circularity (a 2D parameter) is the ratio between the NG2-glia cell body area and its perimeter, indicating how round the cell is, a perfect circle attributed a value of one. By contrast, the solidity, also a 2D parameter, is the ratio between the body cell area and the convex hull area, showing how filled the convex hull area is. Solidity is a parameter that indirectly estimates how thick the cell processes are, or how branched the cell is., and Wwhen the cell body area coincides with the convex hull area, the solidity is one. A significant increase in both the cell body area and the domain area of the two morphological types of NG2-glia was detected relative to the sham cells (Figure 4H,I), although the significance was higher in the case of type II NG2-glia. When the circularity, the cell body volume, the domain volume, and the solidity were compared, these parameters were only significantly higher in type II NG2-glia. Nevertheless, the type I NG2-glia exhibited a tendency towards an increase in those parameters compared to the sham cells (Figure 4J–M).

Total thickness (µm^3^) is a parameter that refers to the volume of all the branches with a threshold of 0.05. In this study, the number of branches included both the major and primary processes, and all the minor branches that derived from them (the secondary, tertiary, and higher order processes) [39]. Interestingly, the average branch thickness (µm^3^) appeared to be the same for type I, type II, and sham NG2-glia morphologies, this being a measure of the relationship between the total thickness of a cell and its number of branches (Figure 4N). Nevertheless, the total thickness and the number of branches was significantly higher in type II NG2-glia and it tended to be higher in type I NG2-glia compared to sham NG2-glia (Figure 4O,P). Thus, NG2-glia not only increase in size and the domain they occupy in response to EAE-induction but also, in the arborization of their processes and their volume, with some thicker branches in type II NG2-glia.

In addition, the Scholl 3D analysis compared the ending Scholl radius (µm), the number of total intersections and the total branch length (µm). The ending Scholl radius is the distance from the cell nucleus to the last concentric circle that surrounds the NG2-glia soma, with a process intersection. The concentric circles of the Scholl analysis have 4 µm intervals in-between them, being the cell nucleus, the center of those circumferences that surround the analyzed cell (Figure 4D) and the number of total intersections measures how many branches cross the concentric Scholl circles. There was a significant increment in cell complexity of both the type I and type II NG2-glia, in both the total number of intersections and total branch length (Figure 4Q,S). This difference with regard the sham cells was more evident in type II NG2-glia. Nevertheless, even if the ending Scholl radius was higher in the EAE groups, there was no tendency for this parameter to increase when the cell changed towards hypertrophy. Our data revealed that in a lesioned scenario, NG2-glia acquire a complex morphology, based on an increment in the total number of intersections (Figure 4Q) in approximately the same Scholl radius (Figure 4R).

Together, our morphological analysis revealed NG2-glia cells in the brain become more complex in response to EAE, also experiencing an increment in size and domain. There was a progressive change in the morphology of NG2-glia in response to lesion, adopting their two morphology types close to the lesion site (type I and II NG2-glia). Type I NG2-glia morphology tended to have a less reactive morphology than type II NG2-glia.

### 3.5. Clonal NG2-Glia Response to EAE Lesion

**Figure 5:**
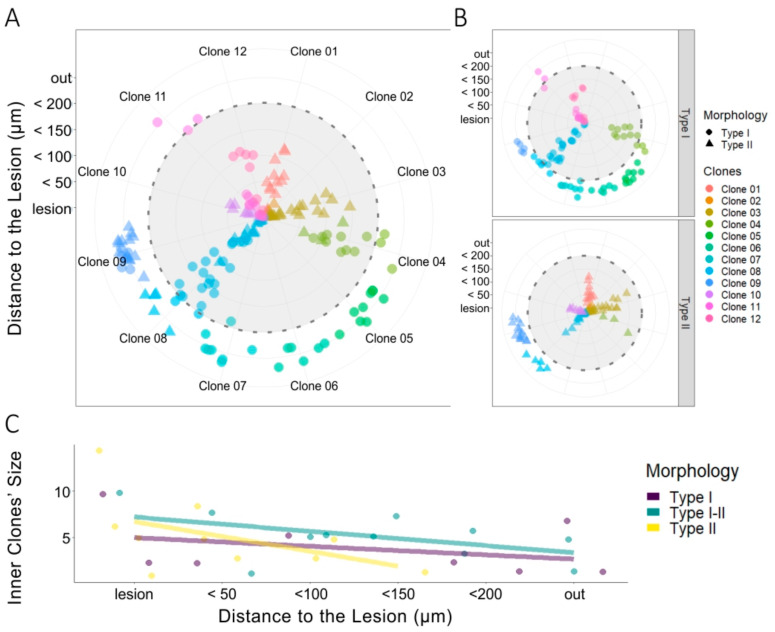
Heterogeneous clonal NG2-glia response to EAE lesions. (**A**) Radial plot of sibling NG2-glia dispersion according to their distance from the EAE-lesion site and the morphology of each cell (*n* = 34). The concentric radii define the lesion core area, with the different perimeters (up to 200 µm) surrounding that area drawn at an interval of 50 µm. Lateral quadrants separate type I (dots) and type II (triangles) NG2-glia into two radial plots. Sibling cells have the same color-code. (**B**) Clonally-related type II NG2-glia inner clones (yellow) accumulate close to the lesion core, within the 150 µm perimeter, and at a higher rate when closer to the lesion core. Type I sibling cells (purple) extended away from the lesion beyond the final 200 µm perimeter and there were a number of sibling cells distributed evenly at all distances from the lesion. Mixed clones of type I and type II sibling cells (green) tend to accumulate closer to the lesion core, although some clones were located outside of the 200 µm perimeter. (**C**) Taken together, type II NG2-glia in inner clones are preferentially located within the 150 µm perimeter and concentrate at a higher rate at the lesion, and closer to it. By contrast, type I cells are dispersed evenly inside and beyond the lesion perimeters established.

Once the morphological parameters of both NG2-glia types had been determined in EAE brains, we analyzed the clonal response in 137 cells from twelve different clones (Figure 5A and Figure 2B) using the perimeters delimiting the infiltrates and lesions (Figure 1B). Activation of these clonally-related cells generated NG2-glia clones of type I, type II, or mixed sibling NG2-glia clones. Those clones formed exclusively by type II NG2-glia were restricted to the lesion core (Figure 5A,B, clones 01–03, 10), whereas type I NG2-glia clones were mainly distributed at a distance from the lesion core (Figure 5A,B: clones 05–07), although some were located close to the lesion core (Figure 5A,B: clones 11, 12). Finally, mixed clones, formed by type I and II NG2-glia were evident at both locations (Figure 5A,B: clones 04, 08 and 09). An analysis of the inner clones showed that sibling cells of type II clones concentrated within the perimeter 150 µm from the infiltrate (Figure 5C), whereas type I NG2-glia sibling cells and mixed clones were dispersed homogeneously from the lesion core (Figure 5C). Thus, while type II cells tended to group around the lesion core, type I cells were distributed more sparsely across the inner to outer regions. Hence, there was a heterogeneous response of clonally-related NG2-glia to lesion.

## 4. Discussion

This study reveals the response of individual NG2-glia progenitors to the damage induced in the EAE mouse model of MS. Using the StarTrack approach in EAE mice, we tracked the progeny of individual embryonic NG2-glia progenitors from the dorsal SVZ in vivo. Labelled sibling NG2-glia dispersed throughout the white and grey matter in a clonal manner. Moreover, a morphometric analysis identified changes in these cells relative to the EAE lesion. Thus, our data revealed that the heterogeneous distribution of NG2-glia in response to EAE lesions is at least in part related to the nature of their progenitors.

### 4.1. Heterogeneity in the Clonal Distribution of NG2-Glia in a Mouse Model of MS

Astrocytes derived from dorsal NPCs in the LV are distributed clonally in either the pia mater, corpus callosum, or cortex [13]. Moreover, sibling astrocytes in the cortex are restricted to become vascular or protoplasmic astrocytes, meaning that their distribution and hence, their function, is determined by the embryonic NPCs [13]. NG2-glia have different temporal and spatial origins during development [28,40]. In particular, the progeny of pallial progenitors migrate to the corpus callosum and cerebral cortex, revealing the relationship between their heterogeneity and their ontogenic origin [31]. Here, we assessed the ten different distributions of NG2-glia clones, showing that specific pallial embryonic progenitors are already committed to a particular clonal distribution in the cortex, pia, corpus callosum, and striatum. As such, clones of NG2-glia derived from embryonic pallial progenitors are more frequently distributed within the lower cortical layers. Furthermore, some sibling NG2-glia may be widely dispersed throughout the striatum, corpus callosum, cerebral cortex, and/or the pia, unlike the astroglial clones [31]. This might imply that NG2-glia heterogeneity is determined early in development. Likewise, this widespread cell dispersion may indicate that they are not limited to act in a small area or that their functions are not so specific.

Clones close to the EAE lesion appear to be larger than those far from it, which could be explained by proliferation and migration towards the lesion. Indeed, NG2-glial cells proliferate in a normal brain [5,14,38] and after brain damage replacing those NG2-glia that die or differentiate into oligodendrocytes [21,41,42,43]. NG2-glia also became hypertrophic and migrate towards the lesion to participate in the formation of the glial scar [21,44]. Moreover, type II NG2-glia clones, corresponding to reactive and hypertrophic cells, tend to accumulate within the lesion core, producing clones with more sibling cells close to the affected area. This indicates that there is a relationship between the distance from the lesion and NG2-glia activation [17,43], and that there is a clonal response. Moreover, while those clones close to the lesion were reactive, some others with sibling cells within the lesion core remained with a non-hypertrophic morphology. Thus, NG2-glial clones are heterogeneous in both their distribution and in their shift towards a reactive cell phenotype after EAE-induction. These different responses of NG2-glia clones not only depend on the molecular environment but also on the progenitor identity, as occurs in astroglial clones in response to EAE injury [19]. NG2-glia could be preferentially connected to each other in a clonal way, similar to the preferential gap junctions between sibling astrocytes [45], which might explain the clonal response of NG2-glia after brain damage. Indeed, NG2-glia share synaptic connections with neurons [7,46] and some connectivity between NG2-glia may exist due to their homeostatic role, and in the control of NG2-glia cell density through active growth and self-retraction of their processes [22,42,43].

Our data reveals that the number of clonally-related NG2-glia cells per clone in the EAE-model is similar to that reported in the same age control mice, although the variance appeared to be higher [13]. Hence, some clones may substitute others, as reported with NG2-glia during development [28].

### 4.2. Heterogenic and Clonal NG2-Glia Responses to EAE Lesions

Central features of the connection between the NG2-glia lineage and their response to EAE lesions were studied here. The different clonal responses to brain damage observed have marked implications in terms of the functional heterogeneity of NG2-glia following brain damage [21,23,24,25,26,27,44], implying that this heterogeneity is established early in embryonic development. Our results reveal that groups of NG2-glia clones display a heterogeneous response to brain damage and perivascular infiltration.

NG2-glia in a 3-nitropropionic acid-lesioned striatum tend to proliferate and accumulate within the perimeter of the lesion [17]. In addition, sibling NG2-glia cells, derived from dorsal progenitors, distribute through the grey and white matter around the lesions, with more clones of NG2-glia close to the lesion than far from it. This suggests that these progenitors may be determined to react upon brain injury and indeed, specific NG2-glia populations proliferate and react upon acute brain injury in both the grey and white matter [46]. Interestingly, endogenous NG2-glia can react to brain demyelination in order to protect axons, maintaining the velocity of axonal transmission with newly-formed but defective myelin [6]. It is crucial to understand the mechanisms regulating the NG2-glia response to demyelination in order to improve remyelination and functional axon recovery. Our results reveal that the NG2-glia response is already determined in their progenitors and therefore, our findings will be of interest for future therapeutic remyelination strategies.

### 4.3. NG2-Glia Activate in Response to EAE Lesions, Undergoing a Progressive Morphological Change to Reactivity Characterized by an Increase in Their Size, Domain, and Arborization

For the first time, we have analyzed morphometric parameters to define the reactive phenotype of NG2-glia in response to EAE lesion. Although some morphological changes of reactive NG2-glia are known [17,21,44], it is unclear how NG2-glia evolves in the face of the severity and hypertrophy in EAE. We found that NG2-glia increase their size, tridimensional non-overlapping domains, or complexity in response to different brain insults [17,44]. By contrast, NG2-glia complexity and size decrease in the first 72 h after ischemia, indicating cell death rather than hypertrophy [45]. These parameters are restored one to two weeks later [45], suggesting that neighboring NG2-glia might maintain cell density and homeostasis [21,47,48].

After injury, NG2-glia undergo a graded continuum of morphological alterations over time and they adopt, at least, two morphologically distinct states as they become reactive: a less severe or non-reactive morphology (type I cells), and a hypertrophic morphology (type II cells). All these changes occur gradually, passing through intermediate stages in between the non-reactive and the hypertrophic NG2-glia. Reactive NG2-glia acquire a rounder shape, thickening their branches to fill their spatial domains and enlarging their individual domain. Interestingly, NG2-glia migrate to the lesion after brain injury and lose their individual domain [21]. Together with their shift to hypertrophy, this change may imply that these cells are involved in the formation of the glial scar after brain damage to restrict the lesion and to promote tissue repair. Moreover, these cells control reactive gliosis [21,26,43]. Reactive NG2-glial cells increment NG2 expression [17,45,49], which is implicated in cell adhesion [50] and associated with a decrease in the differentiation to oligodendrocytes, as well as with an increment in synaptic contacts [51]. Scholl parameters revealed growing complexity in the processes of NG2-glia following activation and hypertrophy, showing that hypertrophied NG2-glia are more branched and complex [17].

Our findings regarding the different clonal response to brain damage have marked implications in understanding the functional heterogeneity of NG2-glia in response to brain damage [21,23,24,25,26,27,44], implying that this heterogeneity is established early in embryonic development. Defining the heterogeneity of NG2 cells in relation to their ontogenic origin is important to address the in vivo reprogramming of NG2-glia to neurons in CNS injury mouse models [52,53,54].

In conclusion, we present relevant features of the NG2-glia lineage in terms of the response to EAE lesions. We demonstrate that NG2-glia from individual progenitors differ in both their spatial fate and in their functional response to EAE lesions.
